# Unique Use of Posterior Sacroiliac Joint Fusion for Pain Relief in Bertolotti Syndrome

**DOI:** 10.1002/ccr3.70810

**Published:** 2025-08-21

**Authors:** Michael Suarez, Shiva Senthilkumar, Mustafa Broachwala, Harman Chopra, Jeffrey L. Chen

**Affiliations:** ^1^ Department of Physical Medicine and Rehabilitation Johns Hopkins University Baltimore Maryland USA; ^2^ College of Medicine University of Toledo Toledo Ohio USA; ^3^ Department of Anesthesiology, Center for Pain Medicine University of California San Diego La Jolla California USA

**Keywords:** Bertolotti syndrome, chronic pain, lumbosacral transitional vertebrae, posterior sacroiliac joint fusion

## Abstract

This case highlights the potential effectiveness of posterior sacroiliac joint fusion in pain relief for patients with Bertolotti syndrome. When a patient presents with pain and lumbosacral transitional vertebrae on imaging, there should be a full evaluation for sacroiliac joint pathology, especially at the joint contralateral to the transitional vertebrae.

## Graphical abstract

Magnetic resonance imaging of bilateral sacroiliac joints. Demonstrating partial sacralization of the left L5 vertebral body with hypertrophic pseudoarticulation with the left sacral ala. Degenerative changes of the right sacroiliac joint are noted.

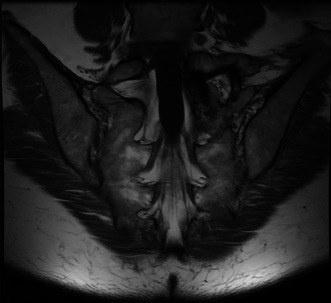

## 1 Introduction

Bertolotti syndrome, first described in 1917 by Italian radiologist Mario Bertolotti, is a symptomatic condition caused by the presence of lumbosacral transitional vertebrae (LSTV) [1]. LSTVs are congenital anomalies of the lumbosacral junction, characterized by the presence of a vertebra with morphological features of both lumbar and sacral vertebrae. These anomalies can manifest as either sacralization of the lowest lumbar vertebra (L5) or lumbarization of the uppermost sacral vertebra (S1). Sacralization occurs when the L5 vertebra fuses with the sacrum, effectively reducing the number of lumbar vertebrae and increasing the number of sacral vertebrae. Conversely, lumbarization involves the S1 vertebra taking on characteristics of a lumbar vertebra, thus increasing the number of lumbar vertebrae and decreasing the number of sacral vertebrae. Both types of LSTVs can present with varying degrees of morphological changes, ranging from broadened transverse processes to complete fusion [2, 3].

LSTVs are a relatively common congenital anomaly, with a prevalence ranging between 10% and 29% in most populations [4]. Most LSTVs are found incidentally on imaging and present asymptomatically. Though when a patient presents with symptoms secondary to an LSTV, it takes on a new term altogether. Bertolotti syndrome is diagnosed when a patient presents with symptoms, such as low back pain, associated with the presence of a LSTV [5]. While the presence of LSTVs are relatively common, the prevalence of Bertolotti syndrome is only about 4%–8%. With that said, it is also thought to be substantially underdiagnosed due to the challenges of its presentation [6].

The hallmark presentation of Bertolotti syndrome is chronic lower back pain that is typically near the lumbosacral junction. Pain can include point tenderness, exacerbation with movement, and/or radiation down the hips and buttocks [6]. The altered biomechanics associated with LSTVs can lead to compensatory stress and pain in the sacroiliac joint (SIJ) as well. The unilateral fusion or articulation between the transverse process of the L5 vertebra and the sacrum or ilium restricts movement on the affected side, leading to increased motion and compensatory stress on the contralateral sacroiliac joint. In fact, according to a study by Illeez et al., the prevalence of SIJ dysfunction in patients with low back pain was notably higher at 28.5% in patients with LSTVs; indicating that patients with Bertolotti syndrome are more likely to experience SIJ dysfunction compared to those without LSTV [7]. Much of the associated back pain with Bertolotti syndrome is likely a multifactorial result of mechanical stress and inflammation from the pseudoarticulation itself, nerve compression leading to a radiculopathy, or degenerative changes in adjacent disc and facets [5]. Confirmation of the diagnosis must be made with radiographic imaging of the lumbosacral junction to investigate the presence of a LSTV after having ruled out other etiologies [1].

The choice of treatment depends on the severity of symptoms, the specific anatomical characteristics of the LSTV, the specific location of pain, and the patient's response to initial conservative measures including physical therapy and activity modification. Common procedural management of Bertolotti syndrome includes injections and/or radiofrequency ablation at the pseudoarticulation site. The most common surgical interventions are resection of the pseudoarticulation or lateral process, and spinal fusion [1]. However, there are instances where treatment guided at the pseudoarticulation site may not be the best choice for the patient. Given the prevalence of SIJ dysfunction in patients with LSTV, it may be worth exploring treatment aimed at the SIJ instead [7]. Here we present a unique case of possibly the first reported use of SIJ fusion for lower back pain associated with Bertolotti syndrome.

## 2 Case History

A 48‐year‐old female with a past medical history of Bertolotti syndrome, confirmed on magnetic resonance imaging (MRI), presented to the pain clinic due to chronic intractable bilateral low back pain. Her diagnosis of SIJ‐mediated low back pain, secondary to Bertolotti syndrome, caused dysfunctional gait, balance, posture, and sleep interference due to its severity. On average, she rated her pain as ranging between 2 and 6/10, and slightly worse on the right side. She also rated the amount of interference her pain had on her quality of life as an 8/10. Her Pain, Enjoyment of Life, and General Activity (PEG) score, which is a three‐item comprehensive pain assessment scale, was an 8/10 [8]. On physical examination, the patient was noted to have positive bilateral FABER, Gaenslen, Compression, and Fortin Finger tests. These positive exam findings highly signified the presence of bilateral SIJ dysfunction. Motor and sensation examination were unremarkable.

Conservative care had been largely unsuccessful for the patient, which included multiple months of physical therapy, bracing, and medication management involving gabapentin 300 mg three times daily, cyclobenzaprine 10 mg nightly, and naproxen 220 mg as needed. Thankfully, she did experience substantial pain relief after receiving multiple steroid injections and radiofrequency ablations targeted at bilateral SIJs. These procedures provide about 80% pain relief on average. The duration of pain relief varied, ranging from 6 weeks to 6 months. While the SIJ injections continued to provide substantial pain relief for weeks, eventually the RFAs provided no relief at all. Given her history of positive results with treatments guided at the SIJs and her desire to get longer lasting pain relief, she was deemed a suitable candidate for posterior approach SIJ fusion/stabilization.

## 3 Methods

**FIGURE 1:**
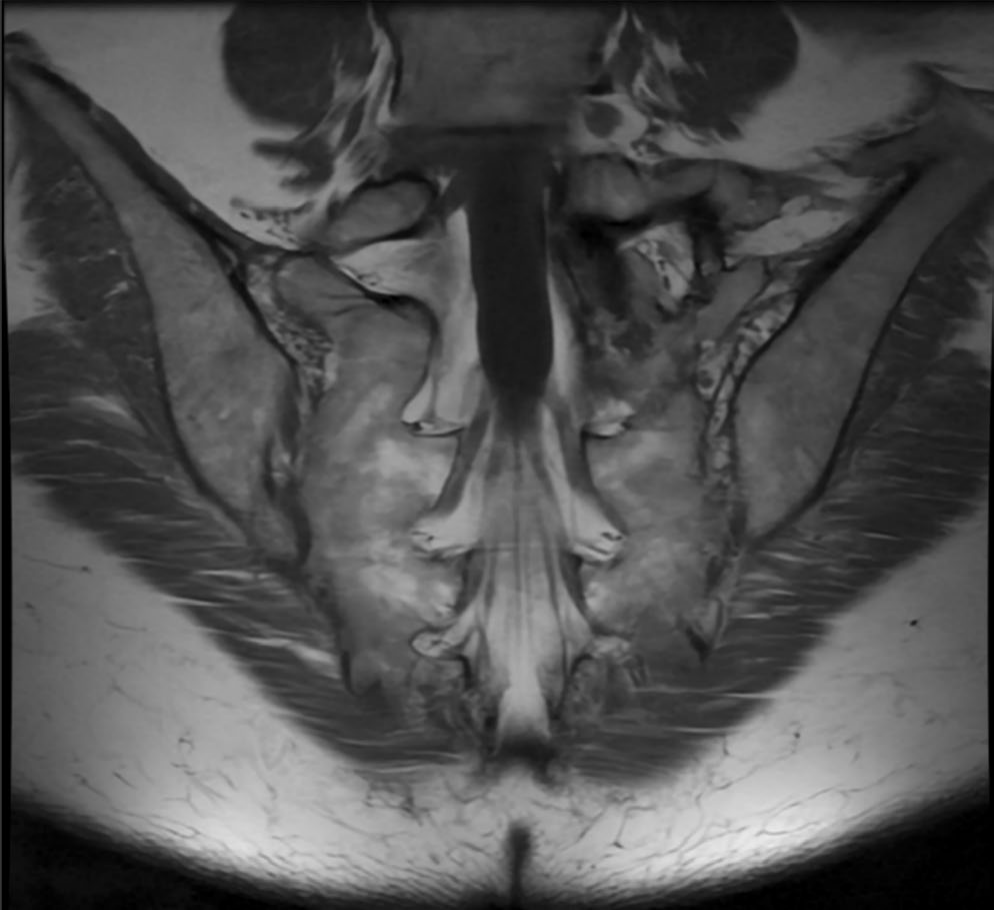
MRI of bilateral sacroiliac joints. Demonstrating partial sacralization of the left L5 vertebral body with hypertrophic pseudoarticulation with the left sacral ala. Degenerative changes of the right sacroiliac joint noted.

In preparation for this procedure, a bilateral SIJ MRI was obtained, demonstrating partial sacralization of the left L5 vertebral body with hypertrophic pseudoarticulation with the left sacral ala and associated degenerative changes at the pseudoarticulation. MRI was also significant for degenerative changes of the right SIJ (Figure 1).

**FIGURE 2:**
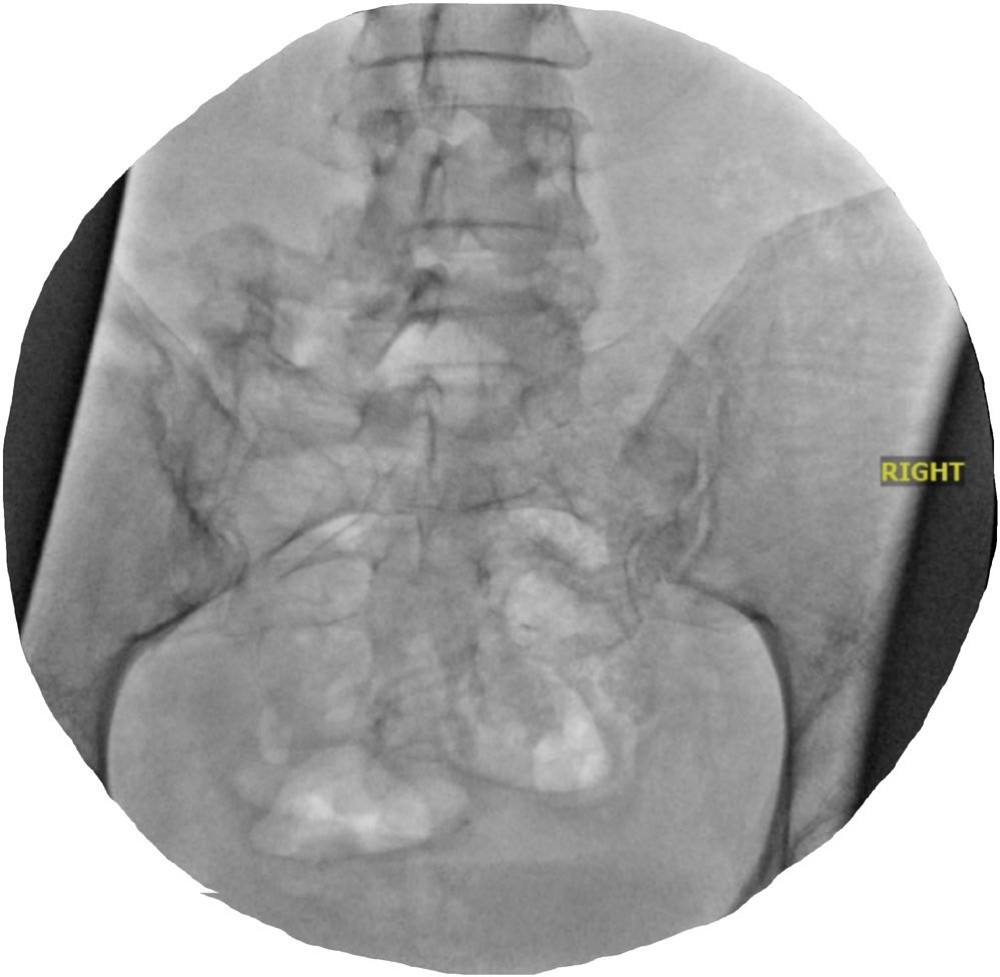
AP radiograph of bilateral sacroiliac joints. Final placement shot of sacroiliac joint stabilizer.

The decision was made to use PainTEQ's LinQ SIJ Stabilization System for this patient's SIJ fusion. Given the LSTV on the left with the patient endorsing slightly worse pain on the right, and imaging demonstrating degenerative changes of the right SIJ, it was conjectured that there was more mobility on the right; thus, a right‐sided placement was decided. The patient eventually underwent a right‐sided fluoroscope‐guided posterior approach SIJ fusion with an intra‐articular placed LinQ allograft implant. The procedure was performed successfully on the day of operation with no associated complications (Figure 2).

## 4 Conclusion and Results

At her 2‐month follow‐up visit, she expressed extreme satisfaction with her outcomes following the procedure. She experienced a 90% improvement in pain and overall quality of life. She reported that her standing posture, gait, and balance improved after the SIJ implant. There was a significant improvement in sleep, with her gaining the ability to sleep throughout the night without pain. She even experienced enough pain relief to allow her to return to her yoga classes, a hobby that she previously loved and consistently participated in. Overall, she stated that this procedure was life‐changing for her. This sentiment and pain relief continued through her 1 year follow‐up.

## 5 Discussion

The SIJ can be a potent source of pain generation in the context of chronic lower back pain, found to be involved in about 14%–22% of patients presenting with chronic lower back pain [9, 10]. When a patient with pain secondary to SIJ dysfunction does not respond well to conservative treatments such as oral analgesics or physical therapy, more invasive interventions are typically trialed. Intra‐articular injections and radiofrequency techniques are common next‐step approaches that are both diagnostic and therapeutic for SIJ pathology. However, due to its recent lack of coverage from no longer being a covered indication, radiofrequency ablation is not an option anymore. The primary target of these interventions is the lateral branches of the dorsal rami of S1–S3 and the dorsal ramus of L5. In some cases, the L4 dorsal ramus may also be included [11]. If the effects of these procedures are diagnostic for an SIJ dysfunction but do not provide long‐lasting pain relief, more advanced options like SIJ fusion can be considered.

Being the largest axial joint in the body, the SIJ plays a critical role in transferring weight and large forces to and from the axial skeleton to the lower extremities [12]. In fact, the SIJ can withstand forces as high as 1440 N or 160 N‐meters without resulting in joint failure [11]. Generally, it is an extremely immobile joint, with two‐thirds of the posterior interface consisting of several ligamentous attachments providing stability, while the anterior portion acts as a true synovial joint [13]. While the SIJ can transmit large bending and compression loads to the lower limbs quite effectively, it intrinsically does not have as much stability in transmitting shearing forces [14]. It has been shown that the transversus abdominis, pelvic floor muscles, and surrounding ligaments play a crucial role in helping resist shearing loads on the SIJ [14]. As such, various injuries and disorders can amplify the tension produced by these structures as the joint becomes less stable, ultimately leading to the generation of pain.

As mentioned earlier, Bertolotti syndrome is characterized by the presence of a LSTV, which can lead to increased forces and altered motion patterns at the SI joint. Specifically, the presence of an LSTV significantly reduces motion at the L5‐S1 level and increases adjacent segment motion [15]. Studies involving lumbar spine fusions have shown that a fusion can in fact lead to increased motion and overall stress across the SIJ [16]. These findings may suggest a reason for the increased prevalence of SIJ pathology in patients with Bertolotti syndrome.

The primary idea behind SIJ fusion as a treatment modality is to restore stability to the joint and reduce abnormal motion, which in turn can decrease the transmission of pathological forces through the joint. Biomechanical studies have shown that posterior SIJ fusion reduces the ROM in flexion‐extension, lateral bending, and axial rotation, thereby stabilizing the joint and decreasing the forces transmitted through it [17]. As a result, by minimizing the mechanical irritation and inflammation of the joint structures, SIJ fusions have been shown to have significant pain reduction compared to conservative management, and are becoming an increasingly more common treatment for low back pain [18].

There have been several advancements in our understanding of the different techniques and approaches of SIJ fusions that have improved the outcomes of this procedure. SIJ fusions can range from open surgeries to minimally invasive procedures; however, at present, the latter is overwhelmingly preferred due to quicker recovery and reduction in complications [19]. There are currently several approaches including lateral, lateral‐oblique, and posterior trajectories that each come with their strengths and weaknesses. The lateral and lateral oblique approaches involve placing two or three screws through the ilium, across the sacroiliac joint, and into the sacrum to stabilize the joint and facilitate fusion. They are the most common approaches and have the most medical literature supporting their use [20]. However, these approaches have been shown to have a higher risk of nerve or vascular injury due to possible breach into the neuroforamen or anterior sacral cortex [9]. Conversely, the posterior approach, which involves placing an allograft within the joint to help promote fusion, utilizes a safer operative corridor for implant placement by remaining within the ilium and sacrum [9]. This reduces the possibility of damaging nearby neurovascular structures, resulting in minimal soft tissue manipulation and blood loss [21]. The posterior approach placed allograft also allows the joint to continue to maintain movement and is not a true fusion.

The SECURE trial, being the largest study on the posterior approach, showed a significant reduction in adverse effects while still providing comparable clinical outcomes compared to the lateral approach [22]. This study showed a total adverse effect rate of 4.3% using 12‐month safety data [22]. The posterior approach also provides unique advantages to biomechanical improvements using the LinQ system. A cadaveric comparative study showed that the posterior approach has similar performance in stabilizing the SIJ during flexion‐extension motions and superior performance in stabilizing the joint during lateral bending and axial rotation motions compared to the lateral approach [23]. This study also showed that unilateral posterior fixation can provide stability of both the ipsilateral and contralateral SI joint [23]. While SIJ fusion systems were initially developed for the lateral approach, the posterior approach has started to gain more attention as a viable technique.

Though the posterior approach results in fewer adverse events compared to other methods, as with all procedures there is still a risk of complication. Though not consistently tracked in studies, the most common complication is nonunion. While infrequently encountered, it can be a concern as it may result in persistent pain and may require additional surgical intervention [24]. Other complications, such as implant migration and implant malposition, are less common complications with reported rates of 0.35% and 0.2%, respectively [11].

To maximize therapeutic benefits and minimize complications in SIJ fusion, selecting appropriate candidates is crucial. Our patient was an excellent candidate, presenting with confirmed SIJ dysfunction secondary to LSTV through examination findings, imaging, and positive pain outcomes from previous SIJ treatments. Additionally, her diagnosis of Bertolotti syndrome provided a clear rationale for her significant SIJ dysfunction. Consequently, she experienced remarkable results from her SIJ fusion, reporting a 90% reduction in pain and an overall improvement in quality of life. This case study highlights a novel application of posterior SIJ fusion in a patient with Bertolotti syndrome. With advancements in SIJ fusion techniques, such as the posterior approach, physicians can offer substantial therapeutic benefits while avoiding many past complications. However, further research is needed to better understand the full spectrum of treatment options, particularly studies involving larger patient groups to assess the effectiveness of unilateral versus bilateral fusion; and to refine our approaches to improve patient outcomes.

## Author Contributions

**Michael Suarez:** writing – original draft, writing – review and editing. **Shiva Senthilkumar:** writing – original draft, writing – review and editing. **Mustafa Broachwala:** resources, supervision, writing – review and editing. **Harman Chopra:** supervision, validation, writing – review and editing. **Jeffrey L. Chen:** conceptualization, funding acquisition, investigation, resources, supervision, writing – review and editing.

## Consent

Written informed consent was obtained from the patient to publish this report in accordance with the journal's patient consent policy.

## Conflicts of Interest

The authors declare no conflicts of interest.

## Data Availability

The data that support the findings of this study are available from the corresponding author upon reasonable request.
